# Aortoiliac Occlusion in a Rare Instance of Leriche Syndrome Type I in a 65-Year-Old Woman With Chronic Leg Discomfort Refractory to Pregabalin

**DOI:** 10.7759/cureus.48858

**Published:** 2023-11-15

**Authors:** Shreya Rani, Anum Khaliq, Syeda Alveera Batool, Muhammad Usman Khan

**Affiliations:** 1 Community Medicine, Indira Gandhi Institute of Medical Sciences, Patna, IND; 2 Community Medicine, King Edward Medical University, Lahore, PAK; 3 Neurology, Division of Neurocritical Care, College of Medicine, University of Florida, Gainesville, USA; 4 Community Medicine, United Medical and Dental College, Karachi, PAK; 5 Cardiology, HCA Houston Healthcare Northwest, Houston, USA

**Keywords:** aorto-occlusive disease, peripheral arterial diseases, cardio vascular disease, atherosclerosis, leriche syndrome

## Abstract

Aortoiliac occlusive disease (AIOD), also known as Leriche syndrome, is a form of peripheral artery disease (PAD) that involves narrowing, and in severe cases, complete occlusion, of infrarenal abdominal aorta and/or iliac and femoropopliteal arteries. It classically presents as a triad of symptoms, i.e., leg pain, erectile dysfunction, and abnormally weak or absent femoral pulses. If untreated, it can progress to ischemia and gangrene of the affected regions of pelvis and lower extremities. Like any other PAD, AIOD is most commonly caused by atherosclerosis and usually occurs in strong association with severe cardiovascular diseases. Due to the rarity of this disease, its incidence and prevalence are still unknown making it harder to diagnose especially in patients without the classic risk factors and typical presentation. We report a case of AIOD in a 65-year-old woman who presented with atypical symptoms. She was diagnosed with AIOD type I upon further investigation, which was managed successfully.

## Introduction

Aortoiliac occlusive disease (AIOD), also referred to as Leriche syndrome, is a rare disorder marked by a triad of claudication, impotence, and diminished/absence of femoral pulses. AIOD was first characterized by Robert Graham in 1914, but it wasn't until a few years later that Henri Leriche referred to the triad of symptoms as a syndrome and gave it his name. Most of the time, this condition is brought on by the persistent development of atherosclerotic plaques, which may then progress to thrombus formation in the aorta, occluding it [1]. As a result, risk factors are the same as those for atherosclerotic disease and include hyperlipidemia, hypertension, male sex, diabetes mellitus, and smoking [2]. The infrarenal abdominal aorta, iliac, and femoropopliteal arteries exhibit atherosclerosis, which contributes to the pathophysiology of Leriche syndrome [3,4]. Effective collateral pathways gradually emerge as the illness progresses [5]. 10% of people with the disease initially show no typical and acute symptoms and acquire collateral circulation as a stenosis of more than 50% is required along with ischemia caused by physical activity or exercise to cause symptoms [6,7]. However, over time, symptoms like chronic claudication and peripheral artery disease (PAD) emerge. Due to vague presentation of the symptoms and lack of awareness of the condition among the general practitioners, exact prevalence and incidence are still unknown. This poses a significant challenge in daily practice. An accurate diagnosis must be made by using diagnostic tools like the ankle-brachial index (ABI), color Doppler ultrasonography, and computed tomography angiography (CTA). Because of the rarity of this disease and misdiagnosis of the condition, this report can be a valuable source of information for other medical professionals that must treat this condition.

## Case presentation

A 65-year-old African American female patient with a past medical history of hypertension, hyperlipidemia, arthritis, vitamin D deficiency and three episodes of distal venous thrombosis (DVT) in her 30s had a tele visit to the cardiovascular clinic for progressive and persistent severe pain in both lower extremities. The patient described the pain to be of intensity 9/10. The pain was worse at night while sleeping and aggravated by activity. The pain was refractory to neuropathic pain medication pregabalin. It was also associated with tingling, altered temperature sensation, and paresthesia. She denied chest pain, shortness of breath (SOB), headache, dizziness, and palpitations. She had a history of multiple back surgeries, breast reduction surgery, and hysterectomy. She has a family history of hypertension. Her brother and sister died of cerebrovascular accident (CVA) in their 60s. She was a former smoker and quit 15 years back and drinks alcohol socially. 

An arterial ultrasound (US) of the lower extremities showed monophasic waveforms throughout both the lower extremities. She had abnormal stress myocardial imaging showing moderate reversible defects in the infero-apical region which might represent ischemia with normal systolic function with a calculated ejection fraction of 52% and no regional wall abnormalities. Her venous US of lower extremities showed abnormally dilated bilateral great saphenous vein (GSV) with prolonged reflux and no DVT. Her EKG had nonspecific ST & T wave changes. Her 2D Echo showed ejection fraction of 55-60% with mild aortic insufficiency (AI) and mild mitral regurgitation (MR).

Her recent laboratory tests before admission were normal for white blood cells, red blood cell, neutrophils, monocytes, eosinophils, basophils, and was low for hemoglobin (11.8 g/dl), hematocrit (35.5%) but the platelets count were elevated (501 × 103/L). The lymphocytes count was mildly elevated (40.9%). She had low potassium (3.5 mmol/L) with normal sodium, chloride, bicarbonate, calcium, magnesium, Glucose, blood urea nitrogen (BUN), creatinine, total protein and albumin. She also has normal bilirubin, ALP, ALT, and AST.

**Figure 1:**
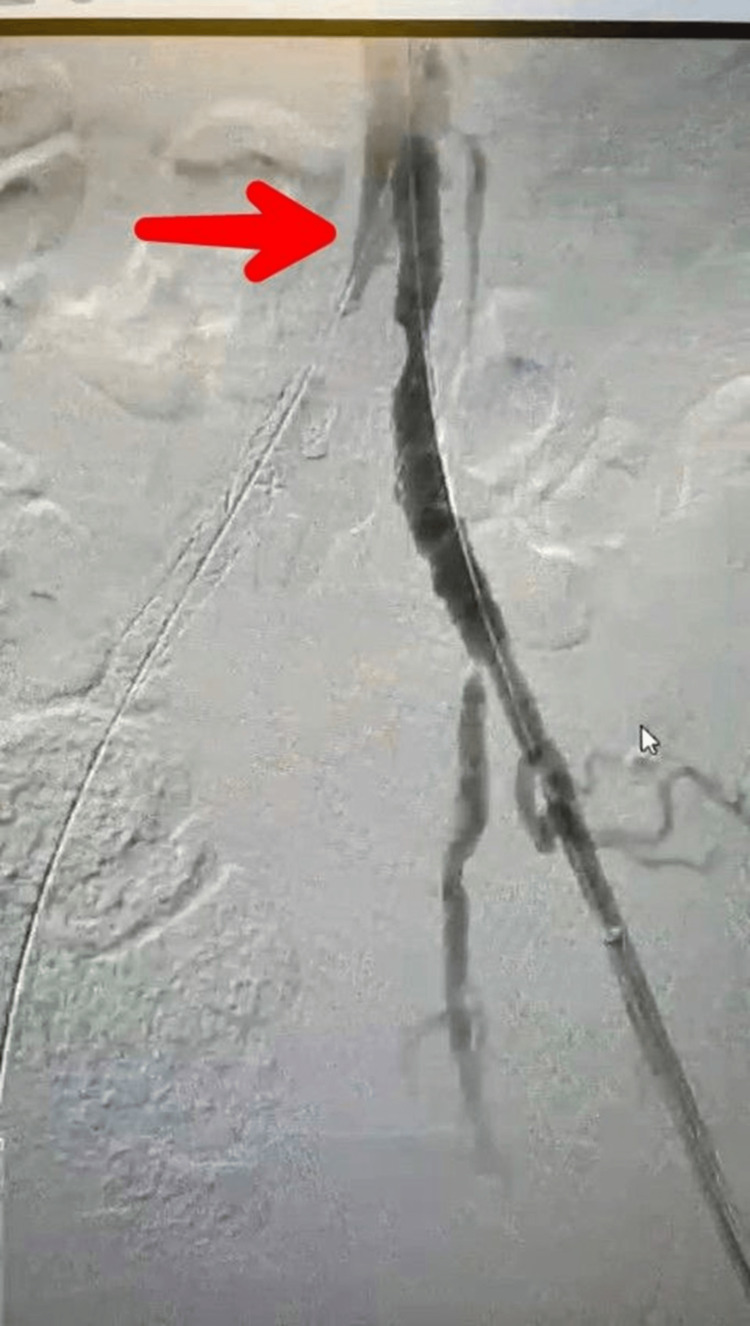
Peripheral angiography, arrow pointing at obstruction at the level of aortic bifurcation with no blood flow to the right iliofemoral arteries and diminished blood flow to the left iliofemoral arteries.

She was admitted the very next day of tele visit and was scheduled for a diagnostic peripheral angiography of lower extremities where no direct blood flow to the right iliofemoral arteries and diminished blood flow to the left iliofemoral arteries with a massive aortoiliac isolated obstruction due to atherothrombosis in the infrarenal abdominal aorta distal to the inferior mesenteric artery (IMA) just above the bifurcation was seen (Figure 1). The blood supply to the lower extremities was by collaterals from the celiac artery, which developed during the course of disease. Based on these findings a diagnosis of Leriche syndrome with type I obstruction (confined to the distal abdominal aorta and common iliac arteries) was made. Based on acuity of the condition, a clinical decision was made and the diagnostic procedure was extended to the immediate clearance of thrombus to prevent ischemia and gangrene.

**Figure 2:**
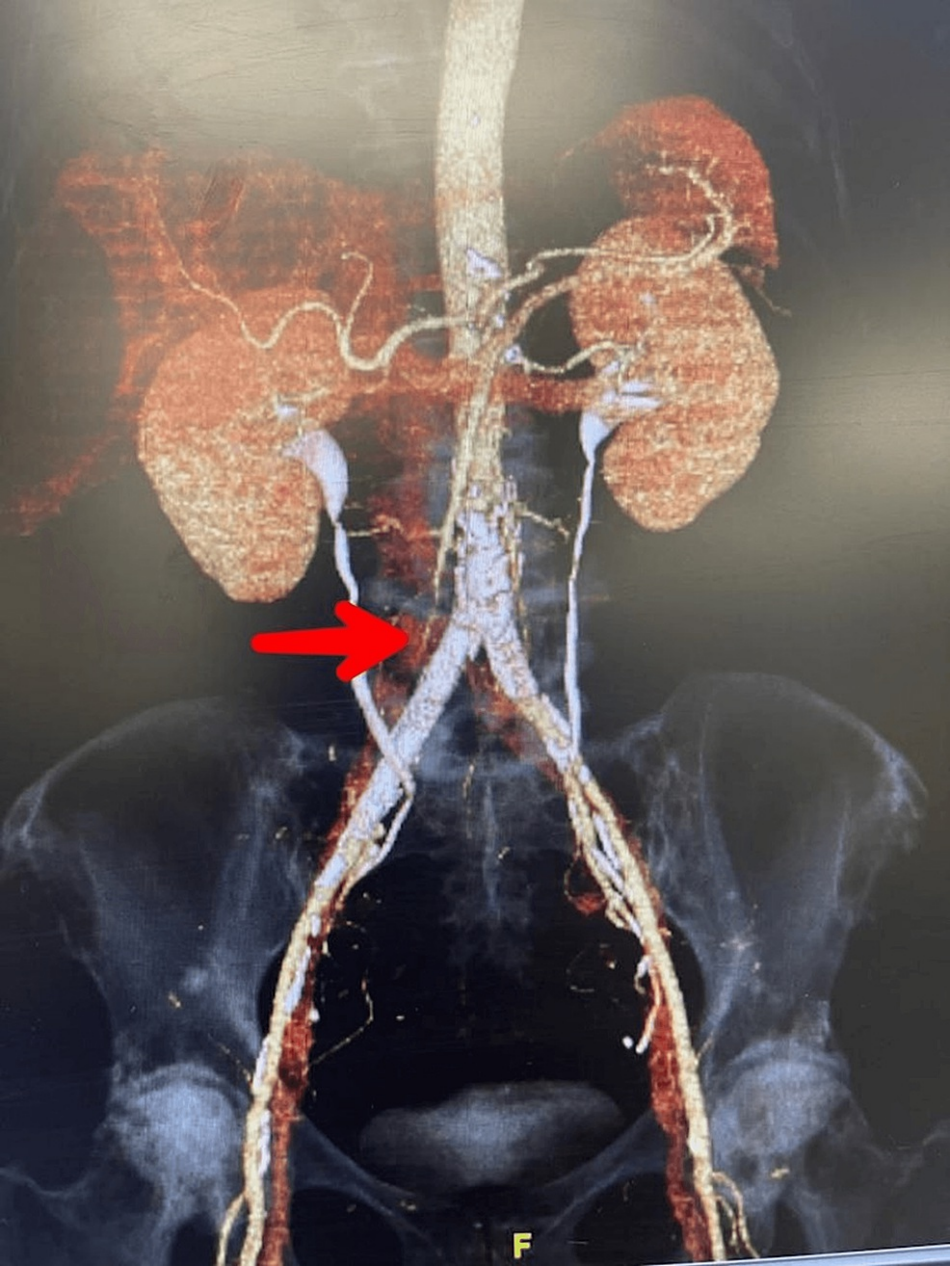
CTA, arrow pointing at diminished blood flow to the iliofemoral arteries CTA: Computed tomography angiography

Then we scheduled the patient for coronary angiography due to abnormal stress test results and risk stratification which was normal where she was found fit in preoperative cardiovascular check up with mild risk. Eventually, she was referred to vascular surgeons who counseled the patient regarding further management possibilities including CTA, by-pass surgery, and aorta angioplasty. CTA done after the peripheral angiography indicated better yet diminished flow through the iliofemoral arteries (Figure 2). About a week later, the aorta angioplasty was performed. Both the common iliac arteries and the right external iliac artery were ballooned and stented. The patient benefited from hospital admission and was sent home on analgesics (non-steroidal anti-inflammatory drugs (NSAIDs), pregabalin), triple anti-hypertensive regimen (amlodipine, thiazide, and losartan), statins, carvedilol, tizanidine, hydroxychloroquine, clopidogrel, and chewable aspirin. The patient was advised to keep follow-up with her vascular surgeons and visit emergency or call 911 in case of recurrence of symptoms. 

## Discussion

The described patient with comorbidities of hypertension and hyperlipidemia was presented for the first time with symptoms consistent with severe bilateral lower limb claudication and was diagnosed with Leriche syndrome with type I obstruction after further investigations. The AOID is classified into three major categories based on the location and size of atherosclerotic occlusions. Type I AOID is limited to the distal abdominal aorta and common iliac arteries; type II AOID is primarily in the distal abdominal aorta with disease spread into the common iliac and external iliac arteries; and type III AOID is more severe and affects the aortoiliac segment and femoropopliteal arteries [8].

Despite the patient having two usual symptoms, the diagnosis in this case report was difficult due to a few factors. First, the patient is a woman, and men are more likely to suffer from PAD including Leriche syndrome. Second, if a high level of clinical suspicion was not maintained, the neuropathic symptoms of tingling, numbness, and paresthesia could result in a misdiagnosis towards neurogenic disease. Neurogenic abnormalities have been shown in a very small number of studies to be a presenting symptom for Leriche syndrome as well [9]. Leriche syndrome can thus delay diagnosis by mimicking relatively benign diseases like sciatica and complex regional pain syndrome [10,11]. Moreover, according to the previous literature, Leriche syndrome is often diagnosed in patients with severe comorbidities such as a history of myocardial infarction, acute pulmonary embolism, dilated cardiomyopathy, and heart failure with reduced ejection fraction [12,13]. Except for hypertension and hyperlipidemia, the patient did not have any severe co-morbidities in this instance. Her coronary angiogram only revealed minimal illness. This highlighted how prompt diagnosis was achieved through appropriate risk stratification and a high level of clinical suspicion. It also made clear that Leriche syndrome should trigger a thorough cardiovascular examination because it may be the first morbidity to appear linked to atherosclerosis.

When it comes to the management, ABI should be used as a first line screening test in patients with suspected AIOD. Although the ABI is typically reduced with significant AIOD, in well-collateralized patients as described in this case report, the ABI value may be normal to only slightly diminished. Among the diagnostic imaging modalities, an arterial US or Doppler US remains inexpensive and non-invasive next best step and was performed in this patient. However, after arterial US, cross-sectional imaging techniques such as CTA and magnetic resonance angiography (MRA) tend to be the next steps followed by pharmacologic or surgical management via open surgical repair or minimally invasive techniques depending upon the extent of the disease [8]. In comparison, based on high degree of clinical suspicion, acuity of the condition, prompt availability of peripheral angiography, and low risk-to-benefit ratio, a peripheral angiography was performed before CTA.

## Conclusions

Leriche syndrome is a rare vascular condition whose incidence and prevalence are not fully known. Due to its unusual clinical presentation and minimal level of clinical suspicion, it is frequently misdiagnosed. Additionally, prompt treatment can protect the patient from fatal morbidities like gangrene and atrophy. This case report will aid doctors in making a diagnosis of the condition, should it show itself in their outpatient clinics or during an emergency, and protect patients from morbidities brought on by inadequate treatment. It also emphasizes the need to consider Leriche syndrome among the differentials when a patient exhibits neurovascular abnormalities of the lower extremities. It also highlights the need for statisticians to make thorough efforts to determine the precise incidence and prevalence of Leriche syndrome.
